# Case Report: Decitabine and venetoclax sequentially followed by FLAG-Ida and venetoclax with immediate allogeneic stem cell transplantation in newly diagnosed acute myeloid leukemia with chromosome 3 inversion/*MECOM* rearrangement

**DOI:** 10.3389/fonc.2025.1530852

**Published:** 2025-03-27

**Authors:** Andrius Žučenka, Milvydė Tamutytė

**Affiliations:** Department of Hematology and Oncology, Institute of Clinical Medicine, Faculty of Medicine, Vilnius University, Vilnius, Lithuania

**Keywords:** acute myeloid leukaemia (AML), allogeneic stem cell transplantation, venetoclax (ABT-199, PubChem CID: 49846579), FLAG-Ida, inv(3)(q21q26.2), MECOM (MDS1 and EV1 complex locus), hypomethylating agents

## Abstract

The prognosis of *MECOM*r AML is poor, with a 5-year overall survival (OS) rate of less than 10%. This is mostly attributable to the low efficacy of all available therapies and high relapse rates even after allogeneic stem cell transplantation (alloSCT), which remains the only curative approach. We report upfront sequential alloSCT with venetoclax-based preconditioning as a safe and effective treatment for two newly diagnosed and fit *MECOM*r AML patients. The sequential alloSCT regimen consisted of triple therapy: preconditioning with decitabine and venetoclax on days 1–5 followed by FLAG-Ida and venetoclax on days 6–10. One or 3 days after preconditioning, the patients underwent busulfan-based myeloablative conditioning and HLA haploidentical or matched related donor stem cell infusion. One month after alloSCT, timely engraftment and complete remission were achieved. At the last follow-up, both patients were in good health and in MRD-negative complete remissions after 11 and 17 months after alloSCT, respectively. The safety and efficacy of upfront sequential alloSCT indicate the need to evaluate this approach for adverse risk of AML in clinical trials.

## Introduction

Ecotropic viral integration site 1 and myelodysplastic syndrome 1 complex rearranged acute myeloid leukemia (*MECOM*r AML) is a rare and distinct entity, accounting for only 1%–1.6% of all newly diagnosed AML cases. The pathogenesis mostly involves a fusion of *GATA2::MECOM (EVI1)* due to inversion or translocation of chromosome 3 [inv(3;3)/t(3;3)] resulting in malignant transcriptomic aberrations; however, more than 120 different chromosomal aberrations involving *MECOM* have been reported (1–6). The prognosis of *MECOM*r AML is poor, with a 5-year overall survival (OS) rate of less than 10% (2, 3, 5, 7, 8). This is mostly attributable to the low efficacy of all available therapies and high relapse rates even in the setting of allogeneic stem cell transplantation (alloSCT), which remains the only curative approach (2, 3, 5, 7–9). Currently, no targeted therapies directly act upon *MECOM* leukemogenic rearrangement. There is a high unmet need for more effective treatments and bridging to alloSCT strategies in this poor-risk genomic subgroup of patients with AML. Herein, we report upfront sequential alloSCT with venetoclax-based preconditioning as a safe and effective treatment for two newly diagnosed *MECOM*r AML patients.

## Methods

Two patients with newly diagnosed *MECOM*r AML underwent sequential alloSCT as frontline therapy. Both patients provided informed consent for data sharing, and the treatment was approved by the Institutional Ethics Committee. We collected baseline characteristics, detailed AML genomics, alloSCT-related parameters, response evaluation, measurable residual disease (MRD) data, overall survival, remission duration, time to neutrophil (ANC) and platelet (PLT) engraftment, grades 3–5 non-hematological toxicity according to the Common Terminology Criteria for Adverse Events (CTCAE) version 5.0

**Figure 1:**
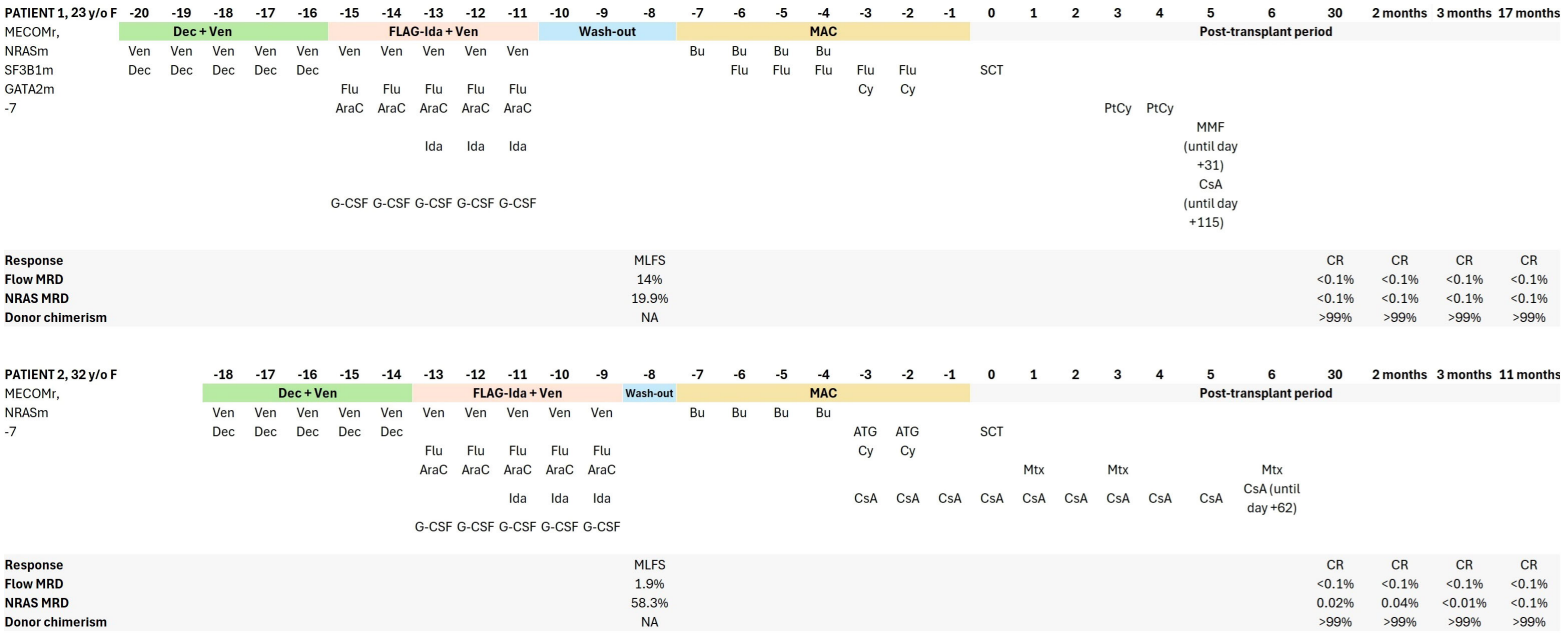
Detailed treatment and response evaluation timeline of patients 1 and 2. F, female; CR, complete remission; MLFS, morphological leukemia-free state; MRD, measurable residual disease; Flu, fludarabine; Dec, decitabine; AraC, cytarabine; Ven, venetoclax; Bu, busulfan; Ida, idarubicin; CsA, cyclosporine A; MMF, mycophenolate mofetil; Cy, cyclophosphamide; PtCy, post-transplant cyclophosphamide; MAC, myeloablative conditioning; ATG, anti-thymocyte globulin; MTX, methotrexate; SCT, stem cell transplantation.

The first part of the frontline sequential triple treatment consisted of cytoreductive hypomethylation/priming with 5-day Dec + Ven (decitabine 20 mg/m^2^ + venetoclax 400 mg/day, on days 1–5). On day 6, FLAG-Ida + Ven was initiated (fludarabine 30 mg/m^2^, cytarabine 2,000 mg/m^2^, G-CSF 5 mcg/kg, and venetoclax 400 mg/day, on days 6–10, idarubicin 8 mg/m^2^, on days 8–10). After a washout period of 1 or 3 days after preconditioning with Dec + Ven and FLAG-Ida + Ven, both patients underwent a 7-day busulfan-based myeloablative conditioning (MAC) and stem cell infusion with standard-of-care graft-versus-host disease (GVHD) prophylaxis and post-transplant care. (**Figure 1**)

## Case description

### Patient 1

A 23-year-old female, who was fit and previously healthy, was diagnosed with *MECOM*r AML in June 2023. The detailed patient characteristics and treatments are summarized in **Table 1** and **Figure 1**. Notably, the patient presented with markedly elevated platelet counts and AML-related diabetes insipidus, both clinical features indicative of *MECOM*r AML. Brain MRI showed infiltration of the infundibulum and absence of the neurohypophyseal signal; however, leukemic cells were not detected in the cerebrospinal fluid. Rapid human leukocyte antigen (HLA) typing identified the patient’s brother as haploidentical; thus, once inv(3)(q21q26.2) was confirmed, preparations for the sequential alloSCT began, and the patient proceeded to the first phase of therapy (Dec + Ven), followed by FLAG-Ida + Ven and MAC. Post-transplant cyclophosphamide (PtCy) was used for T-cell depletion together with cyclosporine A (CsA) and mycophenolate mofetil (MMF)-based GVHD prophylaxis. Pneumonia, sepsis, mucositis, and hand–foot syndrome were reported as grade 3 adverse events during treatment, whereas symptoms of diabetes insipidus have disappeared. Complete remission (CR) with negative MRD and full donor chimerism (99.57%) was confirmed on day +31 after alloSCT. Immunosuppression was tapered and stopped early on day +115 in the absence of GVHD. At the last follow-up (17 months after sequential alloSCT), the patient remained in good health and was in sustained MRD-negative CR with full donor chimerism.

**Table 1:** Baseline characteristics, transplant parameters, and treatment toxicity.

	Patient 1	Patient 2
Sex	Female	Female
Age	19	32
ECOG	0	0
AML-related symptoms	Diabetes insipidus, nausea, fever	Fever, hematomas
AML characteristics		
WBC, ×10e9/L	19.61	28.95
PLT, ×10e9/L	768	485
HgB, g/L	101	99
BM blast count, %	68	72
Dysplasia	Megakaryocytic	Megakaryocytic
FAB classification	M0	M0
FISH	inv(3)(q21.3q26.2)	inv(3)(q21.3q26.2)
SNP-A karyotyping	Monosomy 7	Monosomy 7
NGS	*GATA2* c.959G>A (VAF 38,2% *NRAS* c.182A>G (VAF 35,7%) *SF3B1* c.2225G>A (VAF 33,3%)	*NRAS* c.181C>A (VAF 38.5%)
ELN 2022 risk group	Adverse	Adverse
AlloSCT details		
Donor	Haploidentical sibling	Matched related sibling
Conditioning	MAC (busulfan-based)	MAC (busulfan-based)
T-cell depletion	PtCy*	ATG**
Stem cell source	Peripheral blood	Peripheral blood
CD34+ dose, ×10^6^/kg	8.67	5.96
GVHD prophylaxis	CsA + MMF	CsA + MTX
Treatment-related toxicity		
Grades 3–4 adverse events	Pneumonia, mucositis, sepsis, hand–foot syndrome	Febrile neutropenia, mucositis
Transfer to the ICU	No	No
ANC recovery post alloSCT, days***	16	22
PLT recovery post alloSCT, days***	32	15
ANC recovery from the start of therapy, days	36	40
PLT recovery from the start of therapy, days	52	33
Total length of hospital stay, days	49	37

*PtCy dose: 50 mg/kg on days 3 and 4 post stem cell infusion.

**ATG dose: 2.5 mg/kg on days −3 and −2 prior to stem cell infusion.

***ANC recovery >1 × 10^9^/L, PLT recovery >1 × 100^9^/L.

ECOG, Eastern Cooperative Oncology Group; AML, acute myeloid leukemia; ANC, absolute neutrophil count; PLT, absolute platelet count; HgB, hemoglobin; WBC, white blood cells; AlloSCT, allogeneic stem cell transplantation; ICU, intensive care unit; GVHD, graft-versus-host disease; BM, bone marrow; VAF, variant allelic frequency; MAC, myeloablative conditioning; PtCy, post-transplant cyclophosphamide; ATG, anti-thymocyte globulin; FISH, fluorescence *in situ* hybridization; SNP, single-nucleotide polymorphism array; NGS, next-generation sequencing; CsA, cyclosporine A; MMF, mycophenolate mofetil; MTX, methotrexate; ELN, European Leukemia Network.

### Patient 2

A 32-year-old female, who was fit and previously healthy, was diagnosed with *MECOM*r AML in November 2023. AML-related symptoms included fever and hematomas. Detailed characteristics and treatment details are summarized in **Table 1**, **Figure 1**. Rapid HLA typing identified the patient’s brother as HLA identical; thus, once inv(3)(q21q26.2) was confirmed, sequential alloSCT was scheduled, and the patient proceeded to the first phase of therapy (Dec + Ven), followed by FLAG-Ida + Ven, and MAC. Anti-thymocyte globulin (ATG) was used for T-cell depletion in combination with CsA + methotrexate-based GVHD prophylaxis. The patient experienced treatment-emergent adverse events of febrile neutropenia and mucositis, both of which were evaluated to be grade 3. CR with marginally positive MRD (0.02%) and full donor chimerism (99.5%) was confirmed on day +30 after alloSCT. CsA was tapered and stopped early on day +62 in the absence of GVHD and because of MRD positivity. MRD negativity was confirmed on day +90. At the last follow-up (11 months after sequential alloSCT), the patient remained in good health and sustained MRD-negative CR with full donor chimerism.

## Discussion

We demonstrate two cases of newly diagnosed *MECOM*r AML patients who had the following typical clinical and genomic features of this AML subtype: diabetes insipidus, elevated PLT counts, dysplastic changes in megakaryopoiesis, monosomy 7, and *NRAS*, *GATA2*, *SF3B1* co-mutations (2, 4, 10, 11). Rapid AML genomic testing and HLA typing allowed both patients to successfully undergo an intensive, non-conventional approach of frontline sequential alloSCT using a triple “all-at-once” treatment, which, to the best of our knowledge, had not been previously published.

The rationale for this strategy is mainly based on the poor results of standard available therapies for *MECOM*r AML and the crucial role of alloSCT. In the setting of intensive induction chemotherapy, the reported response rates are only 29%–46% in newly diagnosed patients and less than 20% in the relapsed/refractory (R/R) translating into median overall survival of 5.9–8.4 months only (2, 5, 12). Importantly, alloSCT, especially early in CR1, was associated with significantly better outcomes emphasizing the necessity of fast allotransplantation without exposing the patients to multiple lines of subsequent therapy with possible excessive toxicity and preventing an early relapse (2, 5, 7, 9). Recent reports suggest that the efficacy of hypomethylating agents (HMA) may be similar to intensive chemotherapy with response rates reaching up to 47%, notably lower treatment-related mortality, and comparable survival rates (2, 3, 7). This may be attributed to chemoresistance and the aberrant hypermethylation profile of *MECOM*r leukemic cells (13). The addition of the BCL-2 inhibitor venetoclax to either HMA or intensive chemotherapy in *MECOM*r AML is less described, and available data, albeit scarce, do not seem to demonstrate meaningful improvement in efficacy or survival, which may be explained by the hyperexpression of BCL-XL and high rates of co-mutations in the signaling pathways (*NRAS*, *KRAS*, *PTPN11*) (2, 3, 14–18). Investigational approaches for *MECOM*r AML are currently focused on epigenetic modifiers, inhibition of signaling pathways, and immunotherapy (18).

Based on the aforementioned studies, the first part of our triple therapy consisted of 5-day Dec + Ven to address the aberrant hypermethylation of *MECOM*r AML, provide cytoreduction, and induce epigenetic priming before intensive chemotherapy. The possible efficacy of HMAs as chemosensitizers when used before chemotherapeutic drugs has been previously described in several reports (19–22). After the 5-day Dec + Ven cytoreductive priming on the next day, both patients proceeded to the FLAG-Ida + Ven regimen, which currently demonstrated unprecedented overall response (99%) and MRD negativity (89%) rates in newly diagnosed AML (23, 24). Notably, both patients achieved morphological blast clearance with positive MRD based on bone marrow evaluation during the short washout period of 1 or 3 days and proceeded to myeloablative conditioning and alloSCT.

Myeloablative conditioning during aplasia after Dec + Ven and FLAG-Ida + Ven may have resulted in additional antileukemic efficacy and, most importantly, prevented the risk of leukemia regrowth before engraftment. Furthermore, immediate alloSCT allowed our patients to achieve timely neutrophil recovery, as FLAG-Ida + Ven is associated with prolonged myelosuppression, whereas the addition of Dec + Ven priming to our regimen could have further extended the aplasia period. However, the cumulative high intensity of sequential triple therapy resulted in deep remissions on day 30 after alloSCT in both patients, and the absence of GVHD allowed us to taper and stop immunosuppression early, thus enhancing the graft-versus-leukemia effect, which is of utmost importance in chemoresistant, adverse-risk AML (25, 26). Because of sustained and prolonged MRD-negative CRs in both cases, we did not initiate maintenance therapy with HMAs or prophylactic donor lymphocyte infusions.

The decision to offer immediate alloSCT without achieving a conventional response is supported by experience from multiple reports and a recently published phase 3 ASAP study demonstrating the efficacy and safety of sequential allotransplantation using FLAMSA (fludarabine, cytarabine, amsacrine) followed by standard conditioning regimens in R/R AML patients with active disease (24–35). Implementing upfront sequential alloSCT with FLAMSA and high-dose melphalan as frontline therapy was described in a subgroup of 10 previously untreated secondary AML patients in a single-center study (36). However, the number and outcomes of *MECOM*r AML patients enrolled in the trials were not specified. It is important to emphasize that neither of these studies used HMA and venetoclax in their treatment regimens; however, our recently published single-center experience suggests the clinical applicability of decitabine + venetoclax + cytarabine-based preconditioning before standard conditioning regimens in the sequential alloSCT approach for active high-risk R/R AML (37).

Our case report has several limitations. The treatment was administered outside a clinical trial, and due to the rarity of *MECOM*r AML, we have only described two cases with relatively short follow-up periods. The implementation of sequential HMA + Ven and FLAG-Ida + Ven treatment has not been previously studied, and it remains unknown whether using either of these therapies before sequential alloSCT would have resulted in similar outcomes and whether venetoclax adds efficacy at all in the setting of the *MECOM*r genotype. Furthermore, whether the HMAs should be used before or in combination with chemotherapy to achieve optimal epigenetic priming and possible chemosensitization remains unclear. Despite the absence of life-threatening adverse events in our young and fit patients, high treatment intensity could be one of the major limitations for further clinical evaluation. In addition, the need for rapid HLA typing and donor identification, as well as donor availability, management of stem cell collection, and logistics, may also restrict the timely applicability of this approach despite the transformative effect of post-transplant cyclophosphamide for haploidentical or mismatched donor alloSCT.

Nevertheless, upfront sequential alloSCT treatment was safe and effective in two patients with *MECOM*r AML indicating the need to evaluate this challenging approach for newly diagnosed adverse risk AML in a randomized clinical trial.

## Data Availability

The original contributions presented in the study are included in the article/supplementary material. Further inquiries can be directed to the corresponding author.
